# Mean platelet volume is useful for predicting weaning failure: a retrospective, observational study

**DOI:** 10.1186/s12871-022-01701-w

**Published:** 2022-05-25

**Authors:** Yingying Zheng, Zujin Luo, Zhixin Cao

**Affiliations:** Department of Respiratory and Critical Care Medicine, Beijing Institute of Respiratory Medicine and Beijing Chao-Yang Hospital, Capital Medical University, Beijing, China

**Keywords:** Mean platelet volume, Weaning failure, Invasive mechanical ventilation

## Abstract

**Background:**

To evaluate the usefulness of mean platelet volume (MPV), a marker of inflammation and stress, for predicting weaning failure in patients undergoing invasive mechanical ventilation (IMV) compared to traditional inflammation markers.

**Methods:**

The retrospective observational study including patients who received IMV and underwent spontaneous breathing trial (SBT) was conducted in ICU at Beijing Chao-Yang hospital in China from January, 2013 to December, 2019. According to the weaning outcome, MPV, leukocyte count and C-reaction protein(CRP) were compared between weaning failure and weaning success group. Receiver-operating characteristics (ROC) curves and multivariate logistical regression analysis were constructed to analyze the value of these inflammatory markers for predicting weaning failure.

**Results:**

A total of 261 patients were enrolled in the study and 54 patients (20.7%) experienced weaning failure (45 SBT failure and 9 extubation failure after successful SBT). MPV was a better predictor for weaning failure (AUC 0.777;95%CI, 0.722–0.826) than leukocyte count (AUC 0.6;95%CI,0.538–0.66) and CRP (0.627;95%CI,0.565–0.685). The cutoff value of MPV for predicting weaning failure was 11.3 fl with sensitivity 55.56%, specificity 87.92%, and diagnostic accuracy 81.22%. According to multivariate logistic regression analyses, MPV > 11.3 fl was an independent risk factor for predicting weaning failure.

**Conclusions:**

MPV could be a more valuable marker for predicting weaning failure. and the patients with MPV > 11.3 fl should be attentively evaluated before weaning since they are at high risk of weaning failure, and it would be auspicable for those patients to undergo a noninvasive ventilation or high-flow nasal cannula oxygen therapy after extubation or even an early tracheostomy.

## Background

Invasive mechanical ventilation (IMV) is a procedure widely used for life support in intensive care unit (ICU), however, liberating from IMV is an challenging problem to critical care physicians [1]. The weaning process may account for a significant proportion of total ventilation time, which can delay extubation and lead to complications and/or death [2, 3]. Although a spontaneous breathing trial (SBT) is the major diagnostic test to determine whether patients can be successfully extubated, about 19% of critically ill patients with successful SBT still need reintubation, which is associated with high morbidity and mortality [4]. Hence, it is critical to improve the accuracy of methods predicting weaning outcome.

It has been reported that platelet plays an important role in the procedure of inflammation and immunity [5].Mean platelet volume (MPV) is an simple, easy available and accurate indicator of platelet size and function, and is considered to be a crucial inflammatory marker [6]. In some inflammatory clinical conditions, increasing MPV has been regard as a poor prognostic indicator for patients in respiratory ICU [7]. Among sepsis patients, an increase in mean platelet volume from baseline is associated with mortality in patients with severe sepsis or septic shock [8]. Among influenza pneumonia patients, the change of MVP strongly predicts acute respiratory distress syndrome [9]. It was also reported that higher MPV was an independent risk factors for the poor prognosis of patients with acute pulmonary embolism [10]. Among some patients with surgery, MPV values were significantly higher in the non-survivors following acute abdominal surgery [11], and patients with increased MPV showed lower rates of 1-year survival following undergoing cytoreductive surgery with hyperthermic intraperitoneal chemotherapy [12]. In addition, MPV also has been shown to be a valuable prognostic marker for pulmonary hypertension in chronic obstructive pulmonary disease (COPD) patients [13], coronary artery disease [14], type 2 diabetes mellitus [15].

Critically ill patients are generally in a high state of inflammation, and patients undergoing endotracheal intubation and subsequent IMV show a robust pulmonary inflammation response [16]. Moreover, mechanical ventilation augments preexisting lung injury and inflammation [17]. Besides that, weaning process entails an higher pulmonary and cardiovascular stress among patients who fail to wean than those who are successfully weaned [18]. Hence, weaning failure patients suffer more inflammatory response and stress. However, there were few studies focusing on the relationships between the inflammation or stress and weaning outcome.

Accordingly, we assumed that MPV would be higher in weaning failure patients than in weaning success patients. And we evaluated the effectiveness of MPV for predicting weaning failure in patients receiving IMV, compared to traditional inflammatory markers such as leukocyte counts, levels of C-reactive protein (CRP).

## Methods

### Study design

**Fig. 1:**
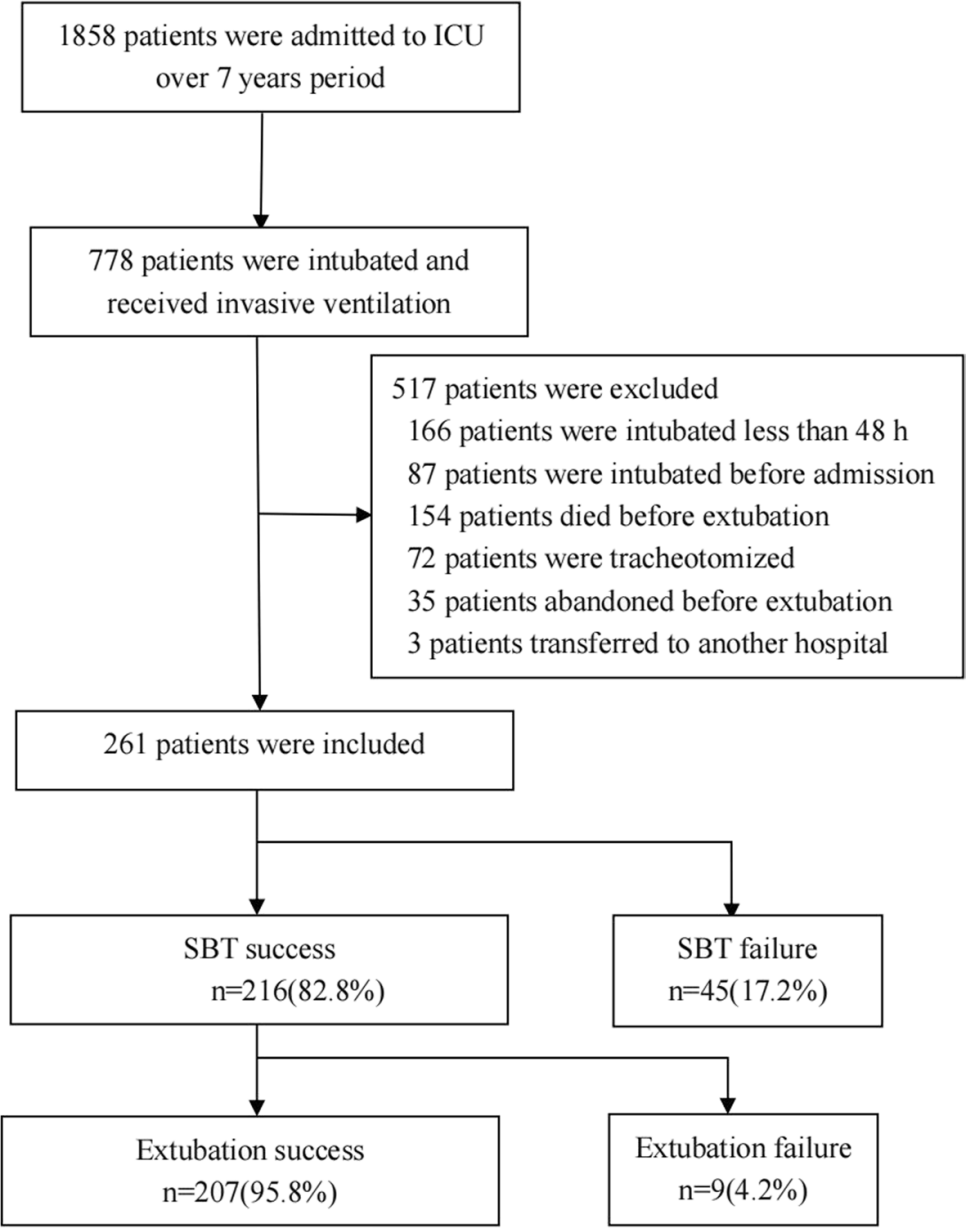
Weaning outcome flow chart. ICU, intensive care unit; SBT, spontaneous breathing trial

The retrospective observational study involving 261 patients receiving IMV admitted to ICU of Beijing Chao-Yang Hospital was conducted from January, 2013 to December, 2019. The weaning outcome flowing chart was shown in Fig. 1.

The ethics committee of the Beijing Chao-Yang Hospital, Capital Medical University approved this study (NO.2020-KE-94). Because of retrospective study, written informed consent was waived by the ethics committee of the Beijing Chao-Yang Hospital, Capital Medical University which gave approval to the verbal consent. All patients were informed by telephone about this research and their consents were obtained, and we keep patient clinical data confidential. All procedures were in accordance with Helsinki Declaration.

### Inclusion criteria

All patients intubated and mechanically ventilated for not less than 48 h were considered eligible for the study if they fulfilled the following: resolution of causes of acute respiratory failure; adequate cough reflex; absence of excessive tracheobronchial secretion; adequate oxygenation (e.g., arterial oxygen saturation > 90% or arterial oxygen tension/fraction of inspired oxygen [PaO_2_/FiO_2_] ≥ 150 mmHg, both on the FiO_2_ of ≤ 0.4 and the positive end-expiratory pressure of ≤ 8 cmH_2_O); adequate ventilatory status (e.g., respiratory rate [RR] ≤ 35 breaths/min with tidal volume ≥ 5 mL/kg of predicted body weight and no significant respiratory acidosis); stable hemodynamics (e.g., heart rate [HR] < 120 beats/min; systolic blood pressure [SBP], 90–160 mmHg; and no or minimal vasopressor use); adequate mentation (e.g., arousable or glasgow coma scale ≥ 13 with no continuous sedative infusions); body temperature < 38 ℃; hemoglobinemia ≥ 80 g/L; and acceptable electrolytes.

### Exclusion criteria

Age < 18 years; pregnancy; tracheotomy or other upper airway disorders; mechanically ventilated less than 48 h; intubated before admission; abandoned before extubation; neuromuscular disease; lack of cooperation; decision to limit active treatment; and refusal of research authorization; incomplete data.

### Weaning protocol

A 2 h-SBT was performed in all eligible patients, which allowed the patients to breathe spontaneously through a T-tube circuit with the FiO_2_ set at the same level used during IMV while the patients were in a semi-recumbent position (45°). The SBT was the first trial for every patient. During the trial, basic vital signs were detected, such as respiratory rate (RR), systolic blood pressure (SBP), heart rate (HR), peripheral oxygen saturation (SpO_2_), five-lead electrocardiographic tracing. Arterial blood gases were analyzed at the beginning and the end of the SBT.

A criteria for SBT failure were: (1)arterial pH < 7.32 with arterial carbon dioxide tension (PaCO_2_) ≥ 10 mmHg higher than baseline; (2)RR > 35 breaths/min or ≥ 50% higher than baseline; (3)peripheral oxygen saturation (SpO_2_) < 90% or PaO_2_ ≤ 60 mmHg at FiO_2_ ≥ 0.4; (4)HR > 140 beats/min or ≥ 20% higher/lower than baseline;(5) SBP > 180 or < 90 mmHg or ≥ 20% higher/lower than baseline; (6)use of accessory respiratory muscles, or thoracic-abdominal paradoxical movement; decreased consciousness, agitation, or diaphoresis. Patients free of these features at the end of SBT were considered to succeed the SBT and subsequently extubated.

Weaning failure was defined as SBT failure or reintubation within 48 h following extubation [20]. Weaning success was defined as extubation successfully and the absence of reintubation for more than 48 h following extubation. We share the same weaning protocol in our department, and this was described in our previous research [19].

### Data collection

Patients’ demographic and baseline characteristics were recorded at enrollment, including age, sex, body mass index (BMI), acute physiology and chronic health evaluation II (APACHE II) score, IMV duration before weaning, comorbidities and acute causes of IMV. Basic vital signs and arterial blood gas were recorded. After extubation, the following was recorded: success or failure of weaning, length of stay (LOS)in ICU, LOS in hospital, ICU mortality, and hospital morality, 28-day survival rate. For patients discharged within 28 days, we followed up their survival status after discharge by telephone.

Complete blood cell counts and CRP at initiation of the SBT were measured. Complete blood cell counts were measured by automatic blood analyzer. CRP concentrations were measured using immunoscatter turbidimetry by Goldsite Aristo (Goldsite, Ltd., China), and the normal value of CRP ranges from 0 to 5 mg/L.

### Statistics

For comparing categorical data, described as frequencies and percentages, Chi square (χ2) test was performed. For Continuous variables, the normal distribution was tested by the Kolmogorov–Smirnov test. Student’s t test was employed for normally distributed data and expressed as mean ± standard deviation. The Mann–Whitney U-test was used for non-normally distributed data and expressed by median (25th-75th percentile). Spearman correlation were used for correlation analysis between MPV and other variables, and the results were displayed as rho and P values. We utilized the receiver-operating characteristic (ROC) analysis to evaluate the sensitivity and specificity of inflammatory markers for predicting weaning failure and to determine the optimum cutoff value for the studied diagnostic markers. Finally, to determine the significant predicting marker of weaning failure, the univariate analysis was used first and then the logistic regression analysis was used to perform a multivariate analysis by a conditional backward stepwise regression model, which resulted in adjusted odds ratios (OR, 95%CI). All analyses were two-tailed, and probability value (p value) less than 0.05 was considered statistically significant. All data were done using SPSS (Statistical Package for the Social Science; SPSS Inc., Chicago, IL, USA) version 22 for Microsoft Windows.

## Results

### Patients’ characteristics and weaning outcome

A total of 261 patients were included in the study, as shown in Fig. 1. Of these, 54 failed the weaning process (45 failed the SBT and 9 extubation failure after successful SBT), 207 succussed the weaning finally. Table 1 shows the baseline characteristics of the patients. Compared to weaning success patients, the failure group had longer IMV duration prior to weaning, higher APACHE II score, lower rates of postoperation. There were significant differences between the two groups in RR, SpO_2_, SBP, PaCO_2_, PaO_2_, FiO_2_, PaO_2_/FiO_2_ (Table 2).

**Table 1:** Baseline characteristics

Characteristics	Weaning failure(*n* = 54)	Weaning success(*n* = 207)	*P*
Age, year (mean ± SD)	73.0 ± 10.9	70.7 ± 13.4	0.261
Male (n, %)	35(64.8)	115(55.6)	0.220
BMI, kg/m.^2^(mean ± SD)	24.8 ± 3.58	24.06 ± 4.73	0.260
APACHE II score on admission (mean ± SD)	21.7 ± 7.0	18.5 ± 6.7	0.004
IMV duration prior to weaning, days (median, 25th–75th percentiles)	6(3–12)	3(2–7)	0.000
Cause of mechanical ventilation, n (%)			
Exacerbation of chronic respiratory disorders	13(24.1)	39(18.8)	0.391
Pneumonia	19(35.2)	58(28.0)	0.304
Sepsis	8(14.8)	30(14.5)	0.952
Postoperation	6(11.1)	61(29.5)	0.006
Congestive heart failure	2(3.7)	5(2.4)	0.637
Neurological disease	4(7.4)	10(4.8)	0.497
others	2(3.7)	4(1.9)	0.276
Comorbidity disease, n (%)			
Hypertension	26(48.1)	118(57)	0.244
Diabetes Mellitus	17(31.5)	57(27.5)	0.567
Chronic heart disorders	18(33.3)	65(31.4)	0.786
Chronic respiratory disorders	13(24.1)	64(30.9)	0.326
Malignancy	15(27.8)	46(22.2)	0.390
Chronic kidney disease	7(13.0)	22(10.6)	0.627
Cerebrovascular disease	8(14.8)	35(16.9)	0.172
Immunosuppression	2(3.7)	2(1.0)	0.190

Continuous variables were presented as median and interquartile range (IQR) or (mean ± standard deviation, SD). Categorical variables were presented as numbers

(n) and percentages (%). Difference of sex, cause of mechanical ventilation, and comorbidity disease between the groups were compared by Chi square (χ2) test. Difference of age, BMI and APACHE II score were compared by Student’s t test. Difference of IMV duration prior to weaning were compared by Mann–Whitney U-test

Other causes of mechanical ventilation included acute pulmonary embolism(*n* = 1) and gastrointestinal bleeding(*n* = 1) in weaning failure group; and cardiac rest(*n* = 1) and gastrointestinal bleeding(*n* = 1) and trauma(*n* = 1) and hepatic encephalopathy(*n* = 1) in weaning success group

*Abbreviation: BMI* Body Mass Index, *IMV* Invasive Mechanical Ventilation, *APACHE II* Acute Physiology and Chronic Health Evaluation II

**Table 2:** Vital signs, arterial blood gas and laboratory findings before weaning

Variable	Weaning failure (*n* = 54)	Weaning success (*n* = 207)	*P*
Vital signs			
RR, breaths/min	21.4 ± 4.49	19.7 ± 4.75	0.020
SpO_2_, %	98(96–100)	99(98–100)	0.003
HR, beats/min	92(79–101)	86(77–97)	0.089
SPB, mmHg	124(112–131)	131(118–144)	0.011
Arterial blood gas			
PH	7.45(7.43–7.47)	7.45(7.45–7.48)	0.914
PaCO_2_, mmHg	41.65(35.52–52.25)	38(34–44)	0.012
PaO_2_, mmHg	85.25(77.3–101.5)	99(81–129)	0.008
PaO_2_/FiO_2_, mmHg	259(212.46–314.25)	302(234–383)	0.003
Laboratory findings			
Leukocyte count, × 10.^9^/L	10.15(8.13–14.45)	8.9(6.7–12)	0.023
Platelet count, × 10.^9^/L	154(112–234)	184(135–253)	0.031
MPV, fl	11.6(10.48–12.88)	10.2(9.5–10.9)	0.000
PDW, %	12(10.98–14.9)	12.1(10.5–13.6)	0.138
Platelet large cell ratio, %	29.8(24.68–38.18)	28.2(21.6–33.5)	0.056
CRP, mg/L	71(33–99)	44(16–86)	0.004

Continuous variables were presented as median and interquartile range (IQR) or (mean ± standard deviation, SD). Difference of RR was compared by Student’s t test. Difference of other variables were compared by Mann–Whitney U-test

*Abbreviation: RR* Respiratory Rate, *HR* Heart Rate, *SBP* Systolic Blood Pressure, *SPO*_*2*_ Peripheral Oxygen saturation, *PaCO*_*2*_ arterial Carbon dioxide tension, *PaO*_*2*_, arterial Oxygen tension, *FiO*_*2*_ Fraction of inspired Oxygen, *MPV* Mean Platelet Volume, *PDW* Platelet Distribution Width, *CRP* C-reactive Protein

### Pre-weaning platelet indices, leukocyte count and CRP

**Fig. 2:**
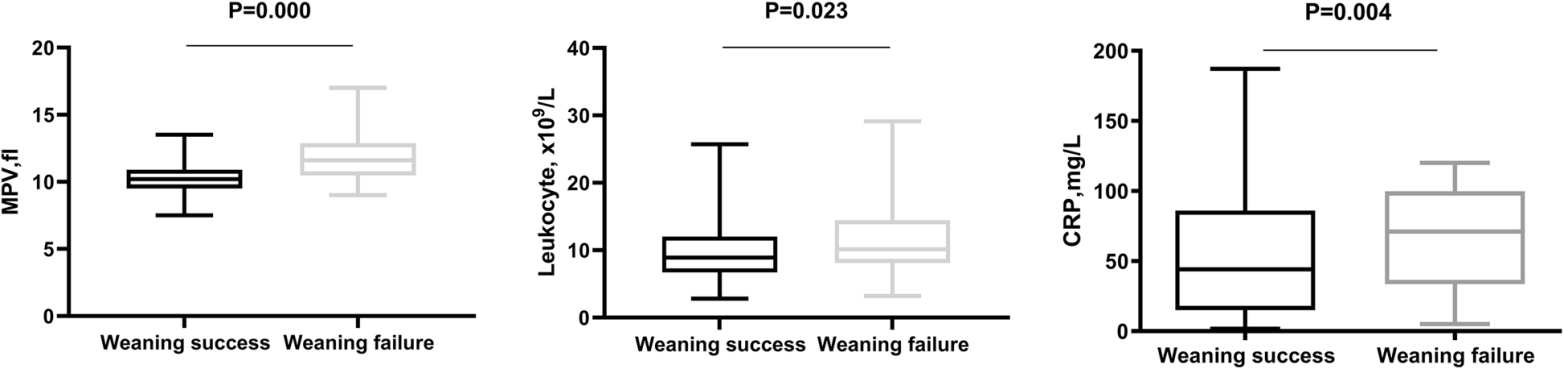
MPV, leukocyte count and CRP before weaning in patients with weaning failure and weaning success

As shown in Table 2 and Fig. 2, leukocyte count(*P* = 0.023), MPV(*P* = 0.000), and CRP(*P* = 0.004) were statistically higher in the weaning failure group than that in the success group. Platelet count was lower in weaning failure group (*P* = 0.031). Platelet large cell ratio tended to be higher in the failure patients, but the p-value did not reach the significance level(*P* = 0.056). There was not significant difference in platelet distribution width (PDW) between the groups. By correlation analysis, MPV was positively correlated with CRP (rho = 0.2, *P* = 0.001) and APACHE II score (rho = 0.197, *P* = 0.001), while it was negatively related with platelet count (rho = -0.246, *P* = 0.000) and PaO_2_/FIO_2_(rho = -0.15*P* = 0.015). However, there was not significant correlation between MPV and leukocyte count (Table 3).

**Table 3:** Correlations between MPV and Leukocyte, CRP, platelet count, APACHE II score and PaO_2_/FiO_2_

	rho	*P*
Leukocyte, × 10.^9^/L	0.044	0.484
CRP, mg/L	0.2	0.001
Platelet count, × 10.^9^/L	-0.246	0.000
APACHE II score	0.197	0.001
PaO_2_/FiO_2_	-0.150	0.015

Spearman correlations were used for correlation analysis, and the results were displayed as correlation coefficients rho and *P* values

*Abbreviation: CRP* C-reactive Protein, *APACHE II* Acute Physiology And Chronic Health Evaluation II, *PaO*_*2*_ arterial Oxygen tension, *FiO*_*2*_ Fraction of Inspired Oxygen

### Predicting ability of MPV, leukocyte count, and CRP level for weaning failure

**Fig. 3:**
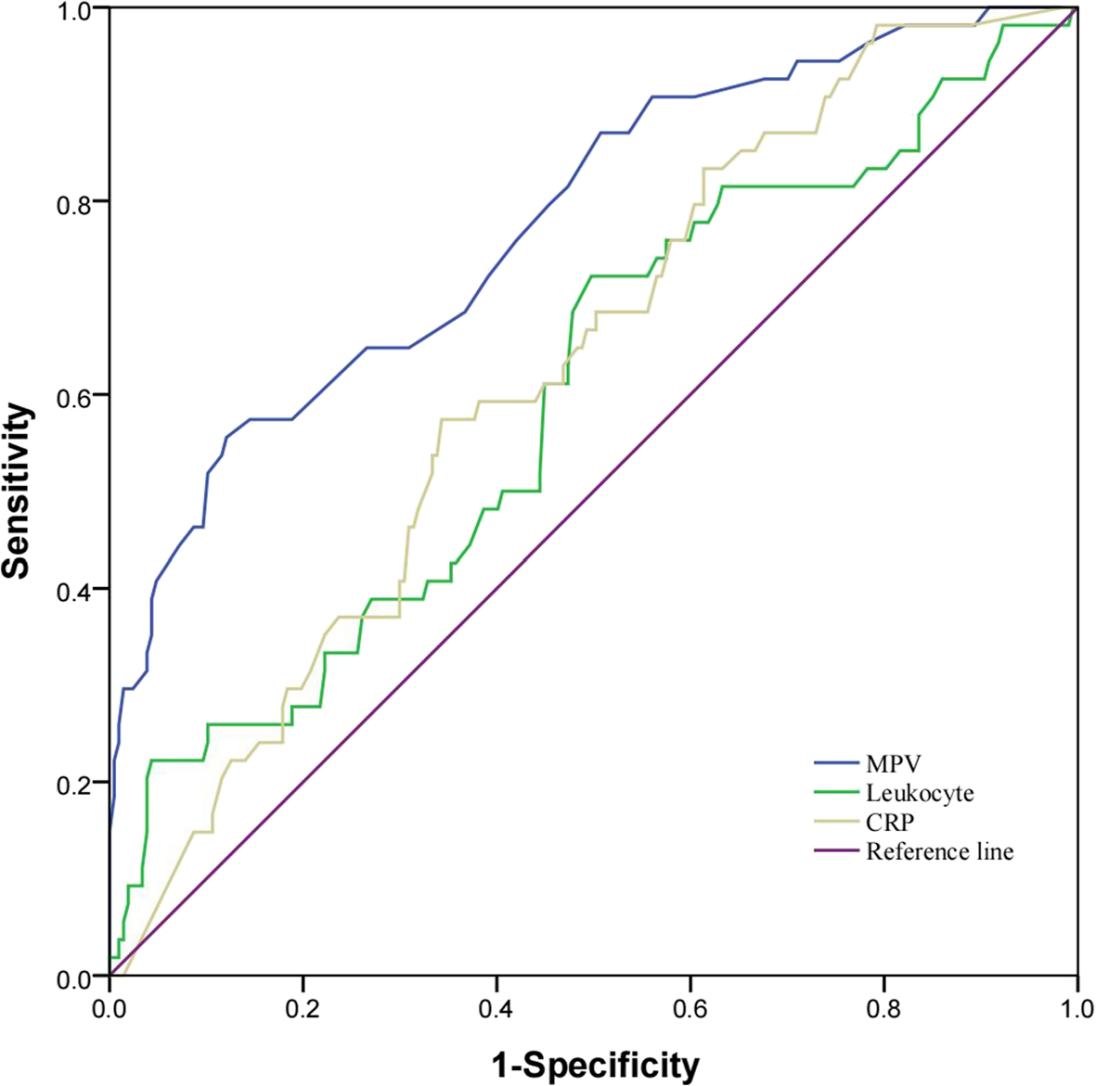
Receiver-operating characteristics curves for MPV, leukocyte count and CRP for predicting weaning failure. MPV, mean platelet volume; CRP, C-reactive protein

The ROC curves of the MPV, leukocyte count, and CRP were shown in Fig. 3. Table 4 showed that the AUC of MPV (0.777;95%CI,0.722–0.826) was higher than that of leukocyte count (0.6;95%CI,0.538–0.66), and CRP (0.627;95%CI,0.565–0.685). The diagnostic accuracy of MPV is highest among the three inflammatory markers.

**Table 4:** ROC curves of MPV, leukocyte and CRP

Characteristics	MPV, fl	Leukocyte, × 10.^9^/L	CRP,mg/L
Cutoff value	> 11.3	> 8.9	> 67
Sensitivity, %	55.56	72.22	57.41
Specificity, %	87.92	50.24	65.7
Positive predictive value, %	54.5	27.5	30.4
Negative predictive value, %	88.3	87.4	85.5
Diagnostic accurary, %	81.22	51.7	63.9
Likehood ratio of positive test	4.6	1.45	1.67
Likehood ratio of negative test	0.51	0.55	0.65
Youden's index	0.43	0.22	0.23
AUC	0.777	0.6	0.627
95% CI	0.722–0.826	0.538 -0.66	0.565–0.685
P	< 0.0001	0.0236	0.0014

*Abbreviation: AUC* Area Under the Curve, *CI* Confidence Interval, *MPV* Mean Platelet Volume, *CRP* C-reactive Protein, *ROC* Receiver-operating Characteristic

The cutoff value for predicting weaning failure was MPV > 11.3 fl, leukocyte count > 8.9 × 10^9^/L, and CRP > 67 mg/L.

According to multivariate logistic regression analyses, both MPV > 11.3 fl and leukocyte count > 8.9 × 10^9^/L were independent factors for predicting weaning failure(Table 5).Multivariate logistic regression was performed with the covariates which showed the *P* < 0.02 by univariate analysis, including APACHE II score, IMV duration prior to SBT, postoperation, pre-weaning PaCO_2_, PaO_2_/FiO_2_, SPO_2_, SBP. Age, sex, and BMI were also included since they often effect prognosis of various diseases [21–23].

**Table 5:** Risk factors for weaning failure

Variable	NO. of failure/total(%)	Univariate analysis			Multivariate analysis		
		Unadjusted OR	95%CI	*P*	Adjusted OR	95%CI	*P*
MPV, fl							
≤ 11.3	24/206(11.7)						
> 11.3	30/55(54.5)	9.1	4.609–17.969	0.000	8.101	3.622–18.117	0.000
Leukocytes, × 10.^9^/L							
≤ 8.9	15/111(13.5)						
> 8.9	39/142(27.5)	2.445	1.364–5.053	0.003	3.495	1.498–8.154	0.004
CRP, mg/L							
≤ 67	23/159(14.5)						
> 67	31/102(30.4)	2.582	1.401–4.756	0.000	2.121	0.987–4.561	0.054

Multivariate logistic regression was performed with covariates including APACHE II score, IMV duration prior to weaning, postoperation, PaCO_2_, PaO_2_/FiO_2_, SPO_2_, SBP, age, sex, and BMI among 261 patients

*Abbreviation:* OR Odds Ratio, *CI* Confidence Interval, *MPV* Mean Platelet Volume, *CRP* C-reactive protein

### Patients’ outcome grouped by MPV cutoff value

Table 6 shows the baseline data, vital signs, arterial blood gas and traditional inflammation markers according to MPV cutoff value. Compared to patients with MPV ≤ 11.3 fl, patients in the group of MPV > 11.3 fl had higher APACHE II score, longer ICU LOS, increased frequency of patients with immunosuppression. Moreover, patients with MPV > 11.3 fl exhibited lower SPO_2_, lower platelet count, higher PDW, higher larger platelet ratio and higher CRP level. The weaning failure rate was higher in patients with MPV > 11.3 fl than in those with MPV ≤ 11.3 fl (Table 5, 30/55(54.5%) vs. 24/206(11.7%), OR = 9.1, *P* = 0.000).

**Table 6:** Patients data according to MPV cutoff value

Characteristics	MPV > 11.3 fl (*n* = 55)	MPV ≤ 11.3 fl (*n* = 206)	*P*
Age, year (mean ± SD)	72.89 ± 11.64	70.79 ± 13.22	0.286
Male (n, %)	36(65.5)	114(55.3)	0.178
BMI, kg/m.^2^(mean ± SD)	24.29 ± 3.68	24.2 ± 4.73	0.894
APACHE II score on admission (mean ± SD)	21.13 ± 6.25	18.69 ± 6.98	0.019
IMV duration prior to weaning, days [median(25th–75th percentiles)]	4(2–9)	3.5(2–7)	0.129
LOS during ICU, days [median(25th–75th percentiles)]	17(9–28)	10(3–20)	0.001
LOS during hospital, days [median(25th–75th percentiles)]	20(15–37)	18(13–28)	0.154
Cause of mechanical ventilation, n (%)			
Exacerbation of chronic respiratory disorders	11(20)	41(19.9)	0.987
Pneumonia	21(38.2)	56(27.2)	0.112
Sepsis	8(14.5)	30(14.6)	0.997
Postoperation	10(18.2)	57(27.7)	0.152
Congestive heart failure	3(5.5)	4(1.9)	0.164
Neurological disease	2(3.6)	12(5.8)	0.741
others	0(0)	6(2.9)	0.348
Comorbidity disease, n (%)			
Hypertension	30(54.5)	114(55.3)	0.916
Diabetes Mellitus	19(34.5)	55(26.7)	0.251
Chronic heart disorders	21(38.2)	62(30.1)	0.253
Chronic respiratory disorders	18(32.7)	59(28.6)	0.555
Malignancy	15(27.3)	46(22.3)	0.442
Chronic kidney disease	7(12.7)	22(10.7)	0.668
Cerebrovascular disease	10(18.2)	33(16.0)	0.701
Immunosuppression	3(5.5)	1(0.5)	0.030
Vital signs			
RR, breaths/min	20(16–24)	20(16–23)	0.526
SpO_2_, %	98(97–100)	99(97.7–100)	0.041
HR, beats/min	86(75–102)	86(78–98)	0.747
SPB, mmHg	129(116–138)	130(118–144)	0.440
Arterial blood gas			
PH	7.45(7.42–7.48)	7.45(7.42–7.48)	0.908
PaCO_2_, mmHg	41.9(35–48)	38(34–45)	0.076
PaO_2_,mmHg	90(76–121)	96(80.8–125.3)	0.162
PaO_2_/FiO_2_, mmHg	265.7(220.6–362.5)	295.3(233.7–295.3)	0.201
Leukocyte count, × 10.^9^/L	9.8(7.6–11.8)	9.1(6.7–12.5)	0.613
Platelet count, × 109/L	152(108–196)	186(140–254)	0.001
PDW, %	15.2(12.7–17.0)	11.8(10.5–12.9)	0.000
Larger platelet ratio, %	38.5(34.6–43.4)	27.1(20.98–31.43)	0.000
CRP, mg/L	72(29–105)	45.5(18–85)	0.025

Continuous variables were presented as median and interquartile range (IQR) or (mean ± standard deviation, SD). Categorical variables were presented as numbers

(n) and percentages (%). Difference of sex, cause of mechanical ventilation, and comorbidity disease between the groups were compared by Chi square (χ2) test. Difference of age, BMI and APACHE II score were compared by Student’s t test. Difference of other variables were compared by Mann–Whitney U-test

*Abbreviation: BMI* Body Mass Index, *APACHE II* Acute Physiology And Chronic Health Evaluation II, *IMV* Invasive Mechanical Ventilation, *LOS* Length Of Stay, *RR* Respiratory Rate, *HR* Heart Rate, *SBP* Systolic Blood Pressure, *SPO*_*2*_ Peripheral Oxygen Saturation, *PaCO*_*2*_ arterial Carbon Dioxide tension, *PaO*_*2*_ arterial Oxygen tension, *FiO*_*2*_ Fraction of Inspired Oxygen, *MPV* Mean Platelet Volume, *PDW* Platelet Distribution Width, *CRP* C-reactive Protein

### Patient outcome

**Fig. 4:**
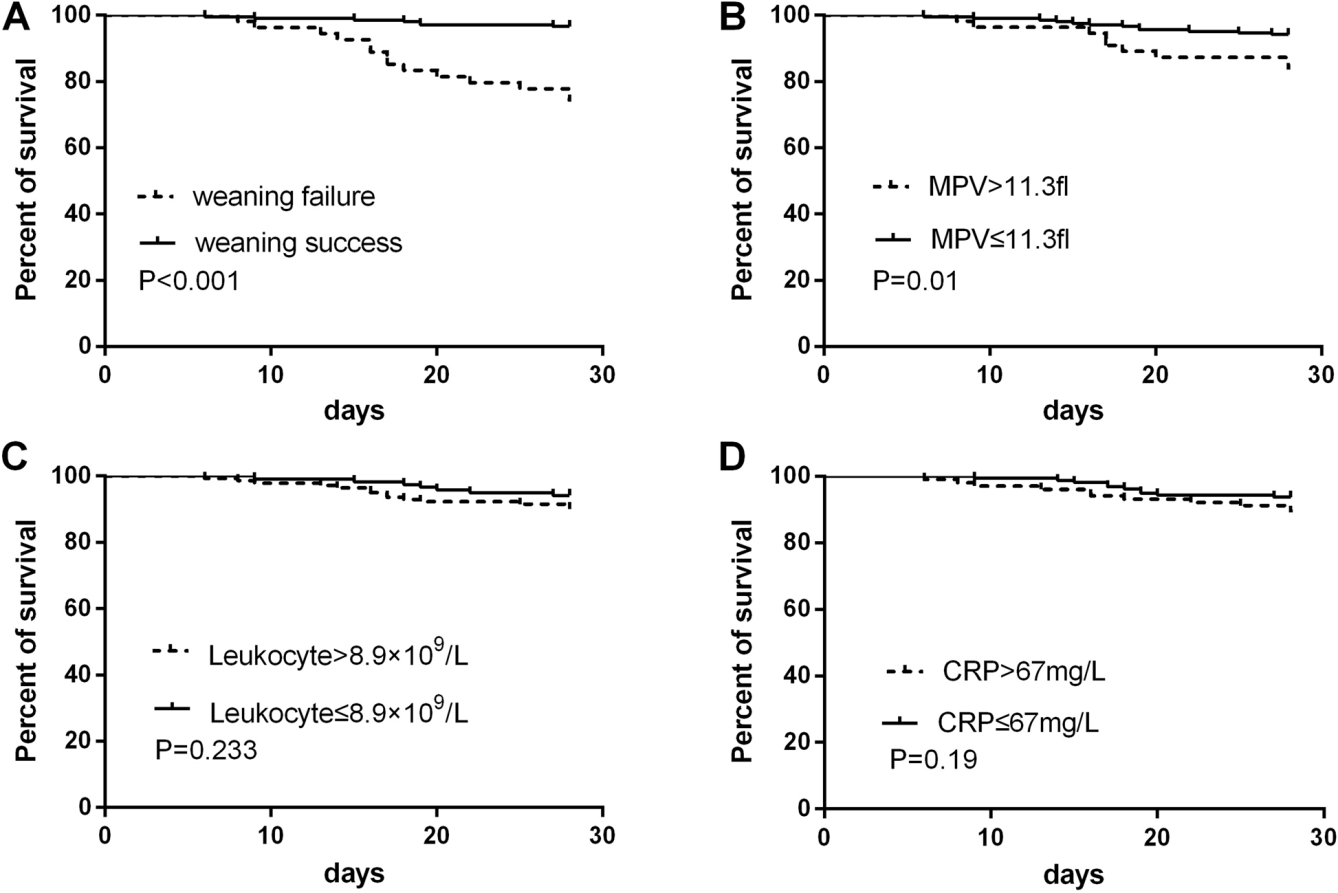
Kaplan–Meier curves for 28-day survival rate grouped by weaning outcome(**A**), MPV(**B**), leukocyte count(**C**) and CRP(**D**). MPV, mean platelet volume; CRP, C-reactive protein

The weaning failure group had higher ICU mortality [11/54(20.4%) vs. 4/207(2%); *P* = 0.000], higher hospital mortality [14/54(25.9%) vs. 7/207(3%); *P* = 0.000], and longer LOS in ICU [18(13–34) vs 10(2–18); *P* = 0.000] compared to successful weaning group. There was not significant difference in LOS in hospital [21(15–34) vs. 19(13–28); *P* = 0.085]. The 28-day survival rate was lower in weaning failure group (*P* = 0.000) and in MPV > 11.3 fl group(*P* = 0.01) by log-rank test, while Leukocyte count (*P* = 0.233) and CRP level (*P* = 0.19) did not significantly affect 28-day survival rate (Fig. 4).

## Discussion

In the current study, we found that MPV > 11.3 fl with the highest AUC of ROC (AUC = 0.777;95%CI,0.722–0.826) was a more valuable marker for predicting weaning failure when compared to traditional inflammatory makers in critically ill patients, and MPV > 11.3 fl was an independent predictor for weaning failure. The odds ratio of MPV > 11.3 fl to predict weaning failure is 8.101, indicating that the risk of weaning failure of patients with MPV > 11.3 fl is much higher than patients with MPV ≤ 11.3 fl.

The underlying mechanisms of rising MPV with weaning failure in critically ill patients remain unclever. The findings may be explained by several possible reasons below. Patients with critical illness often experience more serious status and stronger inflammatory response [16]. Mechanical ventilation can trigger an inflammatory reaction in the lung and subsequent ventilation associated pneumonia or lung injury further enhanced this reaction [17, 24, 25].Moreover, weaning failure patients experienced more pulmonary inflammation response and stress [18].

Hence, the main potential mechanism for higher MPV in patients with weaning failure is severe inflammation and stress caused by unresolved inflammatory diseases and mechanical ventilation. Some studies suggested that system or local infection could increase release of thrombopoietin and different inflammatory cytokines, such as IL-1, IL3 and IL6 and tumor necrosis factor-α, result in increasing thrombopoiesis and lead to the production of younger large platelets in blood circulation [26]. However, the larger platelets function poorly competent, inducing thrombogenic activity and adverse clinical outcomes [26]. In this study, weaning failure patients had higher APACHEII score, longer IMV duration prior to weaning, longer LOS during ICU, higher leukocyte count, CRP level than those in weaning success group, suggesting that the patients of weaning failure were in more serious condition and have a robust inflammatory and stress. Besides that, severe inflammatory status could induce thrombocyte consumption in the peripheral tissue, which finally resulted in the higher MPV [27]. In our study, patients with MPV > 11.3 fl had the lower platelet count than that in patients with MPV ≤ 11.3 fl. Moreover, MPV was inversely related with platelet count among these patients, which was in line with previous study [28]. Hypoxemia may increase the consumption of platelet and promote bone marrow proliferation, which is a possible explanation for increased MPV with lower platelet count in critical patients [7]. Our result suggested that the weaning failure patients had much lower PaO_2_ and PaO_2_/FIO_2_, which could partially explain why MPV was higher in weaning failure group. Taken together, more severe inflammatory, stress and hypoxemia would result in a higher MPV. All of these factors could possibly lead to weaning failure [1]. According, we believe that there was an association between inflammation and stress and weaning failure, and thus MPV might be a more valuable predictor for weaning failure.

Leukocyte level was also an independent predictor of weaning failure in this study, however, by comparing the AUC between MPV and leukocyte count, we concluded that leukocyte was an inferior predictive marker of weaning failure. It is known that sepsis or severe infection could cause either leukocytosis or leukopenia [29]. Many patients presented a normal leukocyte count. Hence, the leukocyte is not likely to reflect disease severity sometimes, thus it may be less valuable to predict weaning failure.

CRP is also the most commonly used metric to investigate inflammation. Nevertheless, we found that MPV outmatched CRP in predicting weaning failure. In line with previous study, CRP was poor for predicting sepsis in children compared to MPV [30].We suggested that CRP is not superior to using MPV for predicting weaning failure.

### Limitations

This study had a few limitations. First, this is a retrospective study in a single center with the relatively small sample, the results of this study ought to be generalized with caution. Second, we did not investigate the medication treatment such as anti-platelet agents and smoking status, which are known to affect MPV [31].Third, certain co-morbidities, nutritional status and anti-inflammatory would affect the inflammatory responses of blood circulation and weaning outcome, but the relevant data were absent since it is difficult to obtain all data in ICU. Fourth, due to the lack of the data, we did not evaluate the correlation of MPV with other more classic parameters related with weaning such as rapid shallow breathing index or the negative inspiratory force, so MPV should be considered together with other traditional clinical and non-clinical parameters to optimize weaning outcome. Fifth, there were a proportion of postsurgical patients who tended to wean successfully, and this possibly cause a bias in the interpretations of the results. Finally, the specific mechanisms of MPV predicting weaning failure cannot be elucidated.

## Conclusions

The ability of MPV to predict weaning failure has not been studied before.

This study indicates that MPV is a preferable marker for predicting weaning failure than traditional inflammatory ones, and MPV > 11.3 fl is an independent predictor for weaning failure. We reveal that patient with MPV > 11.3 fl should be more attentively evaluated before weaning from IMV, and it would be auspicable for those patients to undergo a noninvasive ventilation or high-flow nasal cannula after extubation or even an early tracheostomy. However, further study with a larger sample size is required to confirm this preliminary study.

## Data Availability

The data sets supporting the results of this article are included within the article.

## Abbreviations

MPV: Mean platelet volume
IMV: Invasive mechanical ventilation
ICU: Intensive care unit
SBT: Spontaneous breathing trial
CRP: C-reaction protein
ROC: Receiver-operating characteristics
COPD: Chronic obstructive pulmonary disease
BMI: Body mass index
LOS: Length of stay
APACHE II: Acute physiology and chronic health evaluation II
RR: Respiratory rate
HR: Heart rate
SBP: Systolic blood pressure
SPO_2_,: Peripheral oxygen saturation
PaCO2: Arterial carbon dioxide tension
PaO2: Arterial oxygen tension
FiO2: Fraction of inspired oxygen
MPV: Mean platelet volume
PDW: Platelet distribution width
CRP: C-reactive protein
AUC: Area under the curve
CI: Confidence interval
